# An Integral Model for Target Tracking Based on the Use of a WSN

**DOI:** 10.3390/s130607250

**Published:** 2013-06-03

**Authors:** Carlos T. Calafate, Carlos Lino, Arnoldo Diaz-Ramirez, Juan-Carlos Cano, Pietro Manzoni

**Affiliations:** 1 DISCA, Universitat Politècnica de València, Camino de Vera s/n, Valencia 46022, Spain; E-Mails: jucano@disca.upv.es (J.-C.C.); pmanzoni@disca.upv.es (P.M.); 2 DCS, Instituto Tecnologico de Leon, Guanajuato 37290, Mexico; E-Mail: carloslino@itleon.edu.mx (C.L.R.); 3 DCS, Instituto Tecnologico de Mexicali, Mexicali, Baja California 21396, Mexico; E-Mail: adiaz@itmexicali.edu.mx (A.D.-R.)

**Keywords:** wireless sensor network, intruder tracking and detection, mobile sink

## Abstract

The use of wireless sensor networks (WSN) in tracking applications is growing at a fast pace. In these applications, the sensor nodes discover, monitor and track an event or target object. A significant number of proposals relating the use of WSNs for target tracking have been published to date. However, they either focus on the tracking algorithm or on the communication protocol, and none of them address the problem integrally. In this paper, a comprehensive proposal for target detection and tracking is discussed. We introduce a tracking algorithm to detect and estimate a target location. Moreover, we introduce a low-overhead routing protocol to be used along with our tracking algorithm. The proposed algorithm has low computational complexity and has been designed considering the use of a mobile sink while generating minimal delay and packet loss. We also discuss the results of the evaluation of the proposed algorithms.

## 1. Introduction

Wireless sensor networks (WSN) are comprised of many small devices, called motes, which are deployed over an area of interest in order to detect and monitor events, or to track persons or objects as they move through the sensed area [1]. In surveillance and tracking applications, nodes in a WSN work together to monitor the existence of intruding targets, such as persons or vehicles [2]. Since motes have limited resources, particularly computer power and energy supply, common goals of these applications are reliable detection of targets and fast event notifications with minimal resources consumption.

A WSN monitoring application must periodically collect sensed data and use it to reconstruct the overall status of the monitored area through data aggregation. For this purpose, when the sensor nodes detect an event, they record it and rely on a distributed routing protocol to send the relevant information towards a base station or sink. The sink is a device with much more resources in charge of collecting the information received from the sensor nodes, processing it, and, if necessary, taking the appropriate actions.

It is important that the target events are detected with an acceptable degree of accuracy. To achieve this goal, different algorithms could be used to estimate the intruder location within the sensed area. In addition, the event report should be received by the sink in a very short time, especially for time-critical events. The connectivity issues related to the transmission range of nodes should be under control, and the selected communication protocols must provide a minimum delay and packet loss.

In recent years the research community has proposed several solutions for target tracking in WSNs, focusing mostly on specific elements of the architecture, such as the tracking algorithm or the communications protocol, lacking a holistic solution to the problem. To address this limitation, this paper combines a communication architecture with a tracking algorithm to achieve an efficient intruder detection and tracking system.

The algorithm assumes the use of binary sensors, a mobile sink, and the IEEE 802.15.4 standard for radio communications. We also introduce the Mobile-Sink Routing for Large Grids (MRLG) algorithm, intended to support sink mobility for time-critical applications, for use along with our tracking algorithm. The MRLG routing protocol allows reducing the routing load by relying on local route recovery processes, which provides significant efficiency in scenarios with a large number of sensor nodes. Both algorithms have a low computational complexity.

Many applications may benefit from the use of a mobile sink, among which are target tracking and intrusion detection applications. When an intruder is detected, the sensor nodes report an alarm to the mobile sink, which monitors the progression of the intruder and takes appropriate actions, such as sending the intruder location to the security personnel. The security personnel, endowed with a mobile terminal (sink), may move towards the intruder in an attempt to seize him, using the location feedback provided by the WSN. On the other hand, it has been shown that mobile sinks may improve the WSN lifetime by spreading the transmission overhead of nodes that are close to the sink [3]. However, the main challenge introduced by sink mobility is that the WSN must be continuously and quickly reconfigured to adapt to the topology changes, making sure that information loss is minimal.

The rest of this document is organized as follows. In Section 2 we introduce the previous work. The architecture of the proposed model is discussed in Section 3. In Section 4, we present the proposed intruder tracking algorithm. The MRLG protocol is introduced in Section 5. The performance evaluation strategy is defined in Section 6. The results of the evaluation of the intruder tracking algorithm are discussed in Section 7, whereas the evaluation of the MRLG protocol is reviewed in Section 8. Finally, Section 9 concludes this paper and discusses future work.

## 2. Previous Work

Target tracking based on the use of WSNs has been studied extensively in the last decade [4]. The proposals found in the literature are focused on the application layer and the network layer, but rarely on both. In addition, just a few of them assume sink mobility.

### 2.1. Tracking Algorithms

With respect to the application layer, various approaches have been used in target tracking algorithms. Some are based on different types of measurements, such as the received signal strength (RSS) [5], angle of arrival (AOA) [5,6], time of arrival (TOA) [7], time difference of arrival (TDOA) [8], extended Kalman filters (EKF) [9], and hybrid approaches [10,11], to mention a few. Some others are based on the use of binary sensors, which provide only 1-bit information regarding the presence or absence of a target in their detection area [12].

Concerning proposals using a WSN for target tracking purposes, Li *et al.* [13] addressed the topic of detection and tracking of a single target in a WSN with a static sink, using the coordination between routing protocols and location algorithms. They then extended it to multiple target tracking. In [14], the authors analyzed the fundamental performance limits of tracking a target in a two-dimensional field of binary proximity sensors, determining the accuracy with which a target's trajectory can be tracked. In a later work [15] they extended it to a multiple target tracking solution; in their work they do not address communication protocols used by the sensor nodes, focusing solely on the efficacy of collaborative tracking. Arora *et al.* in [16] studied target tracking and classification. They defined the specifications of WSN deployment for target detection, identifying the best types of sensors to be used by these applications in dense network environments, when focusing on human targets or vehicles. Sheng and Hu proposed a maximum likelihood acoustic source location estimation method for target localization in [17]. However, since this method uses nonlinear optimization, it is difficult to obtain closed form solutions. Chen *et al.* [18] developed an application for control and surveillance in large-scale, real-time WSNs, using a multi-target tracking algorithm, which combined a multisensor fusion method and a Markov chain Monte Carlo Data Association (MCMCDA) algorithm. Their solution is able to automatically start and finish the tracking procedure. The Continuous Object Detection and tracking Algorithm (CODA) was proposed in [19] by Chang *et al.* to detect and track the spread of continuous objects such as wild fires, toxic gases, *etc*. Kim *et al.* introduced a target tracking algorithm based on binary sensors and a static sink. The algorithm utilizes the sensor outputs to estimate individual positions in the path of the target and finds the trajectory that best fits the path points. This estimated trajectory is then used to estimate the current position of the target. Wang *et al.* presented in [11] an approach for target tracking for WSN by combining maximum likelihood estimation and Kalman filtering using the distance measurement. The maximum likelihood estimator is used for prelocalization of the target and measurement conversion. The converted measurement and its associated noise statistics are then used in a standard Kalman filter for recursive update of the target state.

Concerning solutions supporting mobile sinks, Tsaia *et al.* [20] proposed a Dynamical Object Tracking (DOT) algorithm, devised to be used by a mobile source (sink) to chase a moving target. The algorithm uses the knowledge of spatial neighborhood defined on a planar graph, where the face neighbors are identified by a Gabriel Graph. When a target is detected, the sensors need to record the target tracks. When the source requires the target location, it sends a query to the node keeping the track information, which replies with the tracking information, until the source catches the target. Zhang *et al.* [21] proposed a distributed management scheme that uses a set of access points to support transmission from a sensor to the sink. To update the location information, the sink was allowed to choose from different access points while in motion, so that source nodes can be informed about the sink location at all times.

Since energy efficiency is a critical issue in WSNs, several authors have addressed energy-efficient tracking solutions. He *et al.* proposed in [22] a monitoring system for use in military applications, such as a surveillance system, that is able to operate for long periods of time. The system, designed from the standpoint of energy efficiency, was evaluated using a network equipped with 70 MICA2 motes with dual-axis-magnetometers. He *et al.* later developed VigilNet [23], a large-scale real-time WSN system that allows detecting, tracking and classifying targets within a reasonable time, while making efficient use of energy. VigilNet is a system designed for spontaneous military operations in remote areas, where events of interest happen infrequently and with a short duration, such as intruder related events. The VigilNet network infrastructure is based on multi-path diffusion tree rooted at bases. In [12], Cao *et al.* [24] established the relationship between the system parameters and attributes of surveillance, applied to both fixed and moving targets. The authors adopted the model of duty-cycle planning for individual, unsynchronized nodes, allowing nodes to periodically sleep and wake up. In [9], Lin *et al.* introduced an EKF-based distributed adaptive multisensor scheduling scheme for energy efficiency, to improve tracking accuracy. The sensor scheduling problem is formulated as an optimization problem and solved by a sequential three-step heuristic algorithm.

Our proposed tracking algorithm differs from the former ones for several reasons. Since we adopt binary detection sensors to achieve a low-cost solution, we propose a new tracking algorithm based on information aggregation that specifically targets this type of sensors. Moreover, since we want to support mobile sinks to model intruder pursuit by the security personnel, we required a robust and efficient algorithm that quickly adapts to topology changes. To achieve this goal, we devised an algorithm with low computational complexity. Additionally, the previously reviewed proposals are focused on the application layer and do not consider the routing protocol to be used. In contrast, the proposed tracking algorithm relies on our Mobile-sink Routing for Large Grids routing protocol, which provides high efficiency and very low routing overhead in scenarios with a large number of sensors and a mobile sink.

### 2.2. Routing Protocols

Many of the proposed routing protocols that assume sink mobility have been devised for energy efficiency, regardless of the transmission delay [25]. Tong *et al.* proposed the SEnsor Networks with Mobile Agents (SENMA) in [26], a network architecture for low power and large scale sensor networks. In SENMA, mobile agents or sinks are the only receiving terminals in data collections. Thus, when a node detects an event, it must wait for a mobile agent to be in its transmission range to send the collected data, avoiding the need of multi-hop transmission. The Scalable Energy-Efficient Asynchronous Dissemination (SEAD) protocol was introduced in [27] by Kim *et al.*, which assigns specific fixed nodes as the sink's access nodes. The access node is used to represent the moving sink. Sensor nodes deliver data to the access node, which in turn delivers it to the sink without exporting the sink's location information to the rest of the nodes. The protocol assumes that each sensor node is aware of its own geographic location.

MobiRoute [28] is a routing protocol that focuses on scenarios where all the sensor nodes are fixed and have limited energy. Sink mobility is used to increase the network lifetime. The MULE architecture [29] also exploits mobility for energy efficient non-real-time data collection in sparse sensor networks. Song and Hatzinakos proposed the Transmission Scheduling Algorithm Sensor Networks with Mobile Sink (TSA-MSSN) for WSN-based applications with large latency tolerance, minimizing the energy consumption costs. More recently, Rao and Biswas [30] studied the impact of different data collection modes on energy, including network assisted sink navigation.

Concerning those proposals that study sink mobility combined with the IEEE 802.15.4 standard, Chen and Ma [31] conducted a performance evaluation considering mobile sinks and using the AODV routing protocol without major modifications. They considered mainly the delay-energy metric for assessing the performance of different data collection schemes. Vlajic *et al.* [32] presented different strategies for reducing the mobility-related overhead in 802.15.4/ZigBee-based WSNs, assessing the effectiveness of different path-constrained mobile sink trajectories and their suitability in real environments.

Our MRLG algorithm differs from these proposals since for target tracking applications, it is required that the sink receives near real-time feedback about the intruder position in order to pursue him (during short periods of time). Thus, the sink trajectory cannot be constrained. Besides, it does not aim at meeting minimum energy consumption requirements and is thus outside the family of routing protocols that help extending the WSN lifetime.

Recently, the Collection Tree Protocol (CTP) [33] has been proposed for WSNs. CTP is a sink-announcement based protocol where sink announcements are fully propagated throughout the WSN to reach all nodes, allowing the creation of a route tree from all the sensor nodes towards the sink. Any sensor node willing to send or forward a report packet to the sink will consult its routing table to see if there is a valid path towards the sink, and then send the information using that route. In our experiments we compared MRLG against the CTP routing protocol to assess performance gains.

Overall, we find that although the WSN literature is quite broad, only a few routing protocols offer mobile sink support and low-delay traffic delivery [34].

## 3. Architecture of the Proposed Model

The proposed model for target detection and tracking has been devised for scenarios where sink mobility is needed, as in surveillance applications that require a quick response when a target is detected, and real-time feedback of the target location for pursuit purposes. To this end, our proposal is defined as a comprehensive, multi-layer architecture.

Concerning sensor elements, these are motes equipped with low-cost, highly available binary sensors, as Passive InfraRed (PIR) sensors. They are cheap sensors that detect the presence of heat from an object or body nearby. They are also capable of detecting the movement of people through the temperature change caused when moving. In fact, motion detectors usually use PIR sensors.

The communication stack of sensor elements relies on the IEEE 802.15.4 standard, which defines the physical and MAC layers [35]. Since channel congestion is one of the main factors affecting performance in low-bandwidth environments, such as IEEE 802.15.4-based WSNs, the MRLG routing protocol is defined to be used in the network layer of the proposed architecture. MRLG is able to achieve a high performance by minimizing routing overhead when supporting high sink mobility levels. MRLG messages are encapsulated in the data field of IEEE 802.15.4 messages and travel towards the sink.

**Figure 1.**
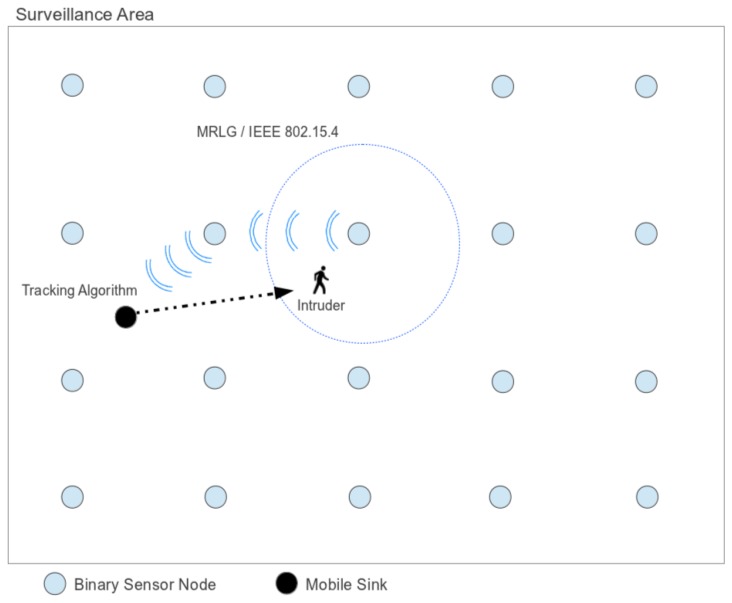
Architecture of the proposed model.

With respect to the sink, it extends the sensor architecture by supporting an application layer on top of the MRLG routing protocol. The application layer is defined through the proposed intruder detection and tracking algorithm. This algorithm has been designed to perform data aggregation efficiently, in order to continuously track the intruder location, while the amount of information sent to the sink is minimal. Figure 1 depicts the architecture of the proposed model.

## 4. Intruder Tracking Algorithm

In intruder detection and tracking applications, a real-time system is actually a soft real-time system, where some latency is allowed [36]. Identifying emergency situations in a few seconds will suffice for satisfying the application requirements. Therefore, applications for intruder detection and tracking require low end-to-end delay values for sensor reports. In the scope of intruder tracking, the challenge is to ensure that the delays from different sources are small enough to allow a timely data fusion, and that the data fusion algorithm is able to provide intruder position estimations as quickly as possible. To meet this goal efficiently, we should have: (i) routing algorithms that are robust, and that quickly adapt to topology changes, while imposing a low routing overhead; and (ii) data fusion algorithms that are able to track the intruder using a small number of reports, avoiding the use of large time windows for report gathering, and also being flexible in the presence of packet loss.

In our approach, the following additional issues should also be taken into consideration:

- The sensor detection is binary within a range of 10 m (equal to the minimum distance between sensors), and sensor coverage is omnidirectional. This implies that, for the proposed grid deployment strategy, the number of sensors simultaneously detecting an intruder can range from one to four.
- The intruder location is constantly updated by the sink node based on the different sensors reports received, and taking into account the timestamp associated with each individual report id.

Algorithm (1) summarizes the process used to continuously estimate the intruder location. This algorithm adopts a report grouping strategy for data fusion purposes that is able to reliably estimate the location of a quickly moving intruder while introducing a low degree of complexity and few calculations. This algorithm was optimized to achieve near real-time position updating for single-target tracking, which was made possible through low computational overhead and incremental filtering based on small time-intervals. Nevertheless, it is worth remarking that multiple-target tracking could be supported in our architecture just by replacing the tracking algorithm with an alternative one, such as [13,15].

Each report is associated with a unique and sequential id by the sink. Notice that each group of data includes several sensor reports, being consecutive groups separated by a time interval (in seconds) defined by the user. Vector $\vec{P_{e}}$ contains the sequence of estimated intruder locations, being constantly updated based on the location associated with the different sensors $\left(\vec{P_{s}}\right)$ that report the intruder's presence. To obtain the sequence of $\vec{P_{e}}$ values, the following operations are performed every time a new sensor report is received:

- Initially (first group created), the intruder location is estimated based on an exponential filter that weights new and old values. Factor α characterizes the behavior of this estimation: higher values make the system more responsive to abrupt changes in the position, whereas lower values make it more conservative.
- From the second group created onward, all report groups are split into micro-groups, and both location $\left(\vec{P_{\text{mgr}}}\right)$ and speed $\left(\vec{V_{\text{mgr}}}\right)$estimations are made for each microgroup. The estimated intruder location is again based on an exponential filter that weights microgroup location estimations $\left(\vec{P_{\text{mgr}}}\right)$ and location estimations derived from speed $\left(\vec{P_{\text{speed}}}\right)$. The latter is calculated as the projection of the previous estimated location $\left(\vec{P_{e}}[\text{last}\_\text{id}-1]\right)$ plus the distance provided by the velocity vector for the time elapsed between the two microgroups.
- Parameter *α* is dynamically calculated, increasing as the time difference between the current time and the initial microgroup time increases. Parameter *β* (*βϵ*[0, 1]) is used to regulate the growth of α, which must never grow beyond its upper bound (*max_alpha ϵ* [0, 1]).

**Algorithm 1** Intruder tracking: position estimation process.
 **input: interval, max_alpha, beta** **begin** set microint = interval/5; #microgroup interval set last_id = 0; set 
$\vec{P_{e}}[0]$ = 
$\vec{P_{s}}[0]$; #initial location equal to the location of the first sensor reporting for each report *id* received do {  if (timestamp[id] - timestamp[0] < $interval) { # for first group only   set 
$\text{alpha}=\frac{\text{timestamp}[\text{id}]-\text{timestamp}[\text{id}-1]}{\text{timestamp}[\text{id}]-\text{timestamp}[0]}\times \text{beta}$   if (alpha > max_alpha) set alpha = max_alpha;   set 
$\vec{P_{e}}[\text{id}]=\text{α}\cdot \vec{P_{s}}[\text{id}-1]+(1-\text{α})\cdot \vec{P_{s}}[\text{id}]$  } else { # group-based estimation   if (timestamp[id]-timestamp[last_-  id+1] < microint) { # microgroup detected    set 
$\vec{P_{\text{mgr}}}$ =    
$\text{average}(\vec{P_{s}}[\text{last}\_\text{id}+1]\;\text{to}\;\vec{P_{s}}[\text{id}])$ #microgroup estimation   } else { # new microgroup    set
$\vec{P_{\text{mgr}}}$ =    
$\vec{P_{s}}[\text{id}]$; #est. group pos. equal to current sensor pos.    set last_id = id - 1;   }   set 
$\vec{V_{\text{mgr}}}$ = estimate_intruder_speed(last_-   id, id) #reports from last_id to id   set
$\vec{P_{\text{speed}}}$ =   
$\vec{P_{e}}[\text{last}\_\text{id}-1]+\vec{V_{\text{mgr}}}\times (\text{timestamp}[\text{id}]-\text{timestamp}[\text{last}\_\text{id}])$   set
$\text{alpha}=\frac{\text{timestamp}[\text{id}]-\text{timestamp}[\text{last}-\text{id}]}{\text{interval}}\times \text{beta}$   if (alpha > max_alpha) set alpha = max_alpha;   set 
$\vec{P_{e}}[\text{id}]=\text{α}\cdot \vec{P_{\text{mgr}}}+(1-\text{α})\cdot \vec{P_{\text{speed}}}$ } } **end**


Finally notice that, for error estimation purposes, the output of this algorithm consists of a sequence of estimated intruder coordinates ( $\vec{P_{e}}$), which are stored alongside with the corresponding timestamp, to be compared against the real sequence of intruder coordinates at the end. This strategy allows determining the tracking accuracy of our solution in any scenario.

## 5. The MRLG Routing Protocol

In this section, we introduce the Mobile-sink Routing for Large Grids (MRLG) algorithm. Although the greatest performance benefits of this routing protocol are more noticeable in large WSNs following a grid topology, it is able to operate in WSNs of any size or topology.

MRLG belongs to the reverse-tree family of protocols, similarly to the CTP protocol [33]. Although both protocols are of the distance-vector type and share a somewhat similar route creation process based on sink announcements, they differ significantly in terms of route costs and, more important, routing maintenance.

Both MRLG and CTP rely on periodic sink broadcasts to announce its presence to neighbor sensors. With CTP, the sink announces its presence with a moderate/large periodicity (typically every 30 seconds), thus being more appropriate for static sinks. With MRLG, the sink announces its presence quite more often, usually every second, to improve responsiveness to mobility. In particular, the procedure followed by MRLG is the following: sensor nodes receiving the sink messages will detect its presence and check whether they were near the sink before. If not, they will update their routing table and notify other sensor nodes about the topology change by propagating the new hop count towards the sink. To avoid too much load, topology update messages will not be propagated throughout the network whenever a node verifies that the uphill node's topology remains the same. Thus, on average, only about half the sensors in the WSN will actually propagate topology update messages, contrarily to CTP where all nodes are involved.

### 5.1. Formal MRLG Description

Our routing protocol is optimized to operate under the following assumptions, which are commonly met in our target scenario: (a) the number of sensor nodes does not increase over time; (b) sensor nodes remain at static positions; and (c) the sink is able to move freely throughout the sensed area, without constraints.

The MRLG protocol distinguishes between three types of neighbor nodes, from the perspective of a particular sensor node: (1) downhill: includes those nodes that are closer to the sink (lower hop count); (2) peers: nodes at the same distance from the sink (similar hop count) and, (3) uphill: nodes further away from the sink (higher hop count). Notice that creating and maintaining the list of downhill, peer and uphill nodes becomes straightforward by listening to the hop count advertised in the messages broadcasted by these nodes, and comparing against the node's own hop count.

The sequence of actions taken by the MRLG routing algorithm when updating routes is the following:

(1) Topology updates are triggered by the sink node. Since the sink can be mobile, it periodically sends Route Request (RREQ) messages to announce its presence at a rate that can be adjusted according to its degree of mobility (one second by default). These messages allow nearby sensor nodes to detect any changes in the sink's position, which may trigger a topology reconfiguration as explained below. The first message generated is fully propagated throughout the WSN, allowing the different sensor nodes to generate a vector field of routes pointing towards the sink node.
(2) Upon listening to the sink's RREQ messages, every sensor node stores the hop count value and the sequence number of the last sink message received; from the set of nodes that share the same (minimal) hop count towards the sink (downhill nodes), it picks one of them as the next-hop for data forwarding. Sensor nodes also store information about other neighbors (both peers and uphill nodes), based on information collected from all overheard messages. In summary, the information stored by each sensor is: <RREQ sequence number, hop count, next_hop, downhill nodes [], peers [], uphill nodes []>.
(3) After processing RREQ messages, sensor nodes will conditionally rebroadcast them taking into account the hop count and the sequence number, according to Algorithm (2). In this algorithm, RREQ is an object that refers to the Route Request Packet, and Node is an object that refers to the data stored by the sensor itself; both objects contain attributes such as hop_count, which refers to the number of hops towards the sink, and seq_number, which is the sequence number generated by the sink (increases every time). Additionally, RREQ contains the ttl attribute, which refers to the Time-to-Live property, and source, which refers to the address of the sensor that broadcasted the packet. Node objects contain methods such as IsUphill(), to determine if a certain node is stored as an the uphill node, and UpdateNeighborList(), which allows updating the information about neighbor nodes by assigning them to the appropriate list: downhill, peers, or uphill.

Differently from the CTP protocol, the possibility of discarding RREQ messages most of the times will bring MRLG significant performance gains by avoiding to introduce routing packets in areas where route updates are unnecessary.

### 5.2. Managing Link Failures

When the link to reach a specific node is lost (after 3 unacknowledged transmission attempts), the routing entry for that node is removed. Afterward, the algorithm will select all the potential next_hop entries from the list of downhill and peer nodes, picking one of the entries with fewest hops to reach the sink. Then, a broadcast message will be generated, informing about the new hop_count and the new next_hop to neighbor nodes. If no valid next_hop neighbors exist, the sensor node inhibits itself from transmitting data until the routes are again refreshed by the sink, to avoid resource wasting.

### 5.3. Long-Term Operation

Since MRLG relies heavily on local route repair mechanisms, broken and depleted sensors could remain undetected for long periods. To avoid this, the sink will periodically generate a special type of RREQ message that must be propagated to all the sensor nodes in the WSN, thereby excluding such sensors from the WSN topology. To maximize the performance, and to keep the routing overhead at very low values, these special RREQs must have an inter-message period much larger than the default RREQ messages.


**Algorithm 2** MRLG route updating: conditional RREQ propagation.
Upon receiving RREQ do {RREQ. hop_count++if (RREQ. seq_number == Node . seq_number) { if (RREQ. hop_count < Node. hop_count) { #improved route to sink  Node . next_hop = RREQ. next_hop  Node . hop_count = RREQ. hop_count  retransmit RREQ after a random delay #to minimize collisions }} elsif (RREQ. seq_number > Node. seq_number) { if (RREQ. hop_count < Node. hop_count) { #improved route to sink Node . next_hop = RREQ. next_hop Node . hop_count = RREQ. hop_count retransmit RREQ after a random delay #to minimize collisions} elsif (Node. IsUphill (RREQ. source)) { #to support field vector reversal Node . next_hop = RREQ. next_hop Node . hop_count = RREQ. hop_count retransmit RREQ after a random delay #to minimize collisions} elsif (RREQ. next_hop != Node. next_hop && RREQ. hop_count == Node. hop_count) { Node . next_hop = RREQ. next_hop RREQ. ttl = 1 # create non−propagating RREQ message retransmit RREQ after a random delay #to minimize collisions} else discard RREQ #no topology changes, drop message}if (RREQ. seq_number == Node . seq_number) { Node . UpdateNeighborList (RREQ. source, RREQ. hop_count)}}


## 6. Performance Evaluation Strategy

To evaluate the accuracy of the proposed intruder tracking algorithm, we carried out a series of simulation experiments where we varied the most critical parameters affecting performance, such as the intruder speed, the intruder trajectory, and the sink speed. Our main output metric is the tracking error, which was calculated by measuring the Euclidean distance between the estimated intruder coordinates and the real intruder coordinates. In this section, we detail the simulation setup issues, along with the tracking error estimation process.

### 6.1. Simulation Setup

To conduct our experiments, we used the well-known ns-2 network simulator. We assumed a mobile sink on each of the test scenarios. The sink, while relying on the sequence of estimated intruder coordinates, will constantly move towards the intruder, simulating a scenario where the security personnel chases the intruder in an attempt to seize him.

The default simulation parameters used in the different experiments are shown in Table 1, unless stated otherwise. We deployed 200 nodes according to a regular grid topology. The distance between sensors was set to 10 m [35]. Since radio communications are based on the IEEE 802.15.4 standard, the transmission range used for all nodes was also set to 10 m. This means that we obtain a connected network (no partitioning effects) with the lowest possible number of sensors. Notice that achieving a connected set with a random sensor deployment would require more sensors for that same area, which would contribute to improve tracking accuracy. Thus, our scenario is a near worst case scenario in terms of tracking accuracy for the sensor communications range defined. The radio propagation model adopted was the two-ray ground. Concerning the sink location, it was initially located on the top left of the scenario. From there, and based on the estimation of the intruder location, it constantly moved towards the intruder.

The routing protocol used in our experiments is MRLG, introduced in Section 5.

The methodology used to conduct the tests was the following: first, we generated the intruder mobility pattern along the monitored area. Then, we calculated the time instant when different sensors were triggered due to the intruder proximity. Since we adopted binary detection sensors, each sensor sent an intruder detected report to the sink upon detection. If the intruder remained within the sensor's detection area, the latter continued reporting the intruder's presence to the sink every 5 s. Based on the different intruder reports gathered, the sink periodically estimated the current intruder location, and dynamically adapted its target, linearly moving towards the last estimated location.

### 6.2. Tracking Error Estimation

**Figure 2.**
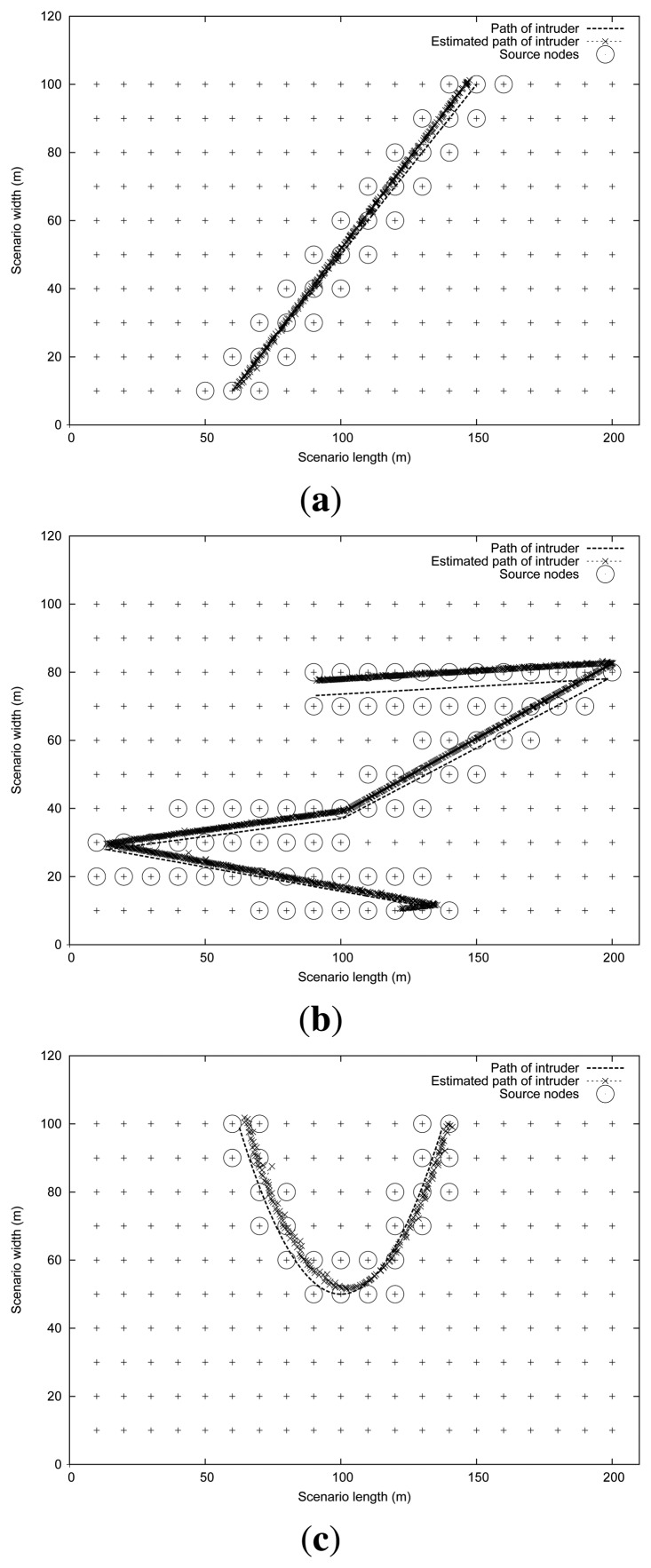
Illustration of the intruder tracking accuracy for different mobility patterns: (**a**) straight, (**b**) random and (**c**) curve.

In order to evaluate the proposed algorithm and to assess its tracking accuracy, we devised a set of intruder mobility patterns (straight, random and curve), which are illustrated in Figure 2. We can observe in Figure 2, besides the intruder's path (represented as a slim dotted trajectory), some circles that highlight those sensors nodes that are activated at some instant of time, generating the appropriate reports for the sink. Crosses represent the sequence of estimations about the intruder's path made by the sink. Differences between real and estimated intruder trajectories are slightly more noticeable in Figure 2(b).

To obtain an accurate measure of the overall error in determining the intruder's trajectory, the following actions are taken:

1. Retrieve the exact intruder location at all times, based on the mobility pattern defined as input to the simulation.
2. Based on the intruder reports received at the sink, filter all signals, keeping only those that correspond to the sensors that have detected an intruder. The available data are: the number of sensors that detected the intruder, the location of each sensor, and the intruder report time advertised by each sensor.
3. For each report received, obtain a new estimate for the intruder's location by relying on Algorithm (1), which creates information groups by combining the reports received at time intervals of fixed duration.
4. Calculate the mean value of the Euclidean distance between the sequence of known intruder coordinates and the intruder trajectory estimation during the entire test period.

In our evaluations, the values adopted for the different tracking algorithm parameters were: *interval* = 5 s, *max_alfa* = 0.25, and β = 0.4. Notice that, as shown in Figure 2, the filters and estimation techniques used in our proposed algorithm are able to achieve a noticeable performance in terms of trajectory estimation, even though simple binary sensors are used.

Concerning the calculated error, notice that three different factors are combined: (i) the intruder location inaccuracy associated with the binary reporting provided by the sensors, which will typically be equal to 10 meters for the first report generated, since this is the sensor's detection range; (ii) the mean estimation error introduced by the chosen data aggregation algorithm, based on the information received; and (iii) the delay experienced by the different reports, when traveling throughout the WSN, until the sink is reached.

## 7. Evaluation of the Intruder Tracking Algorithm

In this section we evaluate the proposed WSN intruder tracking solution. In particular, we start by evaluating the impact of intruder mobility patterns, and then we analyze the impact of sink mobility. Our output metric is the position estimation error, which provides a measure of the average tracking accuracy by determining the mean value between the real and the estimated intruder positions throughout the simulation time.

### 7.1. Impact of the Intruder Mobility Pattern

In this set of experiments we focus on the impact of the intruder mobility pattern and the intruder speed on tracking accuracy. With this purpose we perform tests using the three different intruder mobility patterns shown in Figure 2: straight, random and curve. The intruder speed is varied between 1 and 7 m/s.

**Figure 3.**
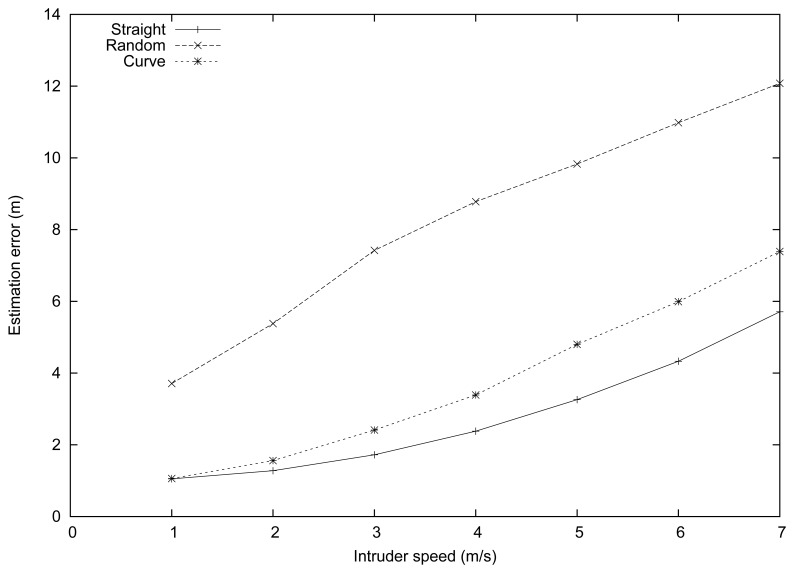
Mean position estimation error for different mobility patterns when varying the intruder speed.

Figure 3 shows the performance results obtained in this set of experiments. In general, the estimation error increased as the intruder speeds varied from 1 to 7 m/s. This is expected since the time required for the reports to travel to the sink, as well as the delays introduced by the report grouping algorithm, are prone to make location estimations less reliable at higher speeds.

Comparing the different intruder mobility patterns, we found that the lowest error in the estimation was achieved when the intruder moved according to a straight path, with error values ranging from 1 to 6 m. The curve and random mobility patterns introduced greater estimation errors (up to 7.4 and 11.9 m, respectively). This is expected since the proposed intruder tracking algorithm makes linear motion assumptions for each microgroup period (see Section 4). Thus, completely random motion patterns are worst case scenarios for our intruder tracking algorithm, which explains the differences detected. Nevertheless, notice that at typical speeds (in the interval [1,3] m/s), the error is maintained reasonably low, and also that the system allows intruder pursuit to take place without much inconvenience in all cases.

### 7.2. Impact of the Sink Mobility

In this set of simulation experiments, we analyze the behavior of the WSN when varying the sink speed. Notice that, in our experiments, the sink used the intruder location estimations in order to continuously move towards the intruder. However, higher sink mobility levels required a faster adaptation of the network topology. Also, the intruder mobility pattern was intimately related to the sink mobility pattern. Thus, our purpose was to determine how these parameters affected the tracking error.

**Figure 4.**
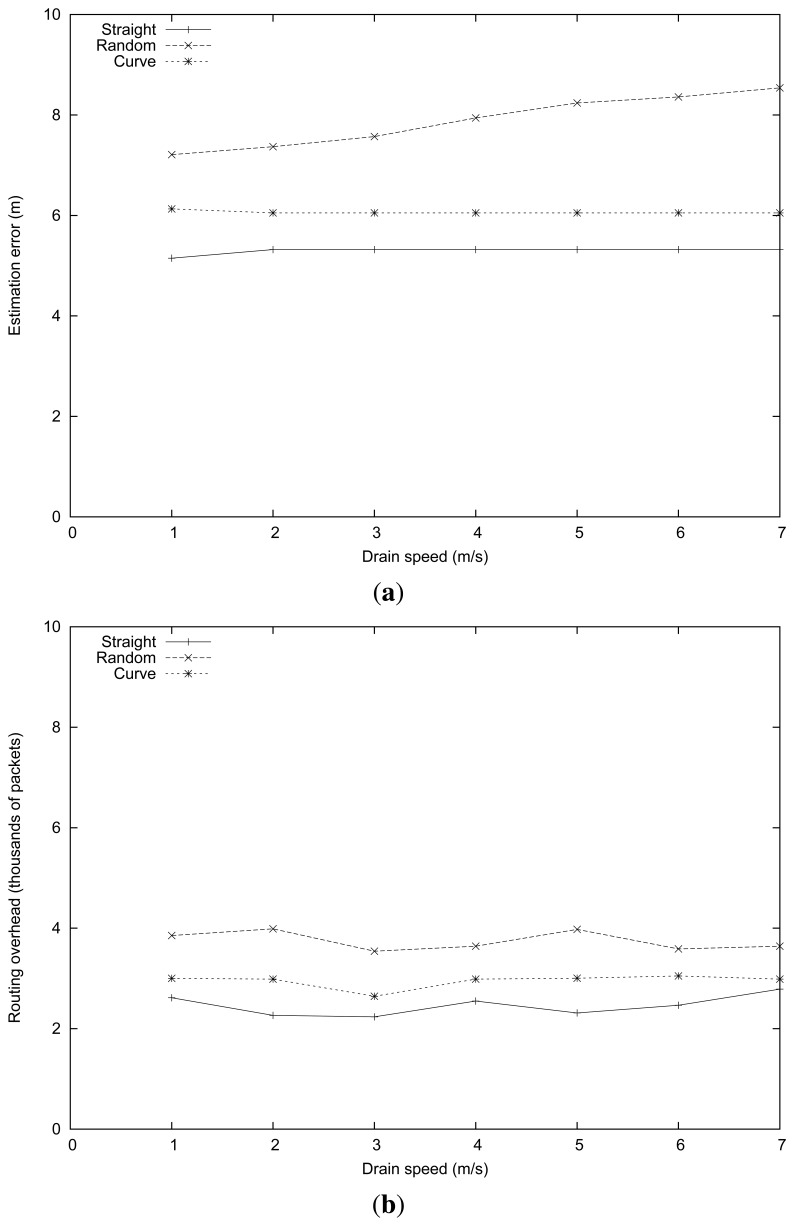
For different mobility patterns when varying the sink speed: (**a**) mean position estimation error, (**b**) routing overhead.

The parameters used in this series of simulation experiments were similar to those of the previous section, but now fixing the intruder speed at 4 m/s and varying the sink speed instead. Figure 4 presents the obtained results. It shows that the routing algorithm was quite robust in the presence of sink mobility, being that the estimated error for the straight and curve mobility patterns is kept stable, with error values of about 5 and 6 m (see Figure 4(a)). For the random path, the estimated error increased slightly (from 7 m to almost 9 m) when the sink speed increased. To understand this difference, notice that the greater error associated with the intruder location estimation makes the trajectory followed by the sink slightly irregular, thus causing more topology changes and more routing-related losses.

In terms of routing overhead, we found that it depends more heavily on the intruder mobility pattern than on the sink speed. In particular, more irregular intruder mobility patterns (in this case, the random pattern) are associated with more routing updates. Notice that the sink mobility pattern is intimately related to the intruder's mobility pattern, which explains the phenomenon observed. In addition, we found that the MRLG routing protocol is highly efficient at managing high sink mobility levels, being that the routing overhead barely varies for high sink speeds.

## 8. Evaluation of the MRLG Protocol

In this section, we assess the improvements introduced by MRLG compared with CTP (see Section 2). To evaluate the performance of the MRLG protocol under the IEEE 802.15.4 technology, we conducted a series of simulation experiments using the network simulator ns-2 [37]. The methodology adopted to conduct the tests was the following: each obtained value is the average of ten independent experiments, providing in most cases a confidence interval smaller than 5% for a confidence level of 95%. Each of the test scenarios adopts a grid topology for the sensor nodes and has a mobile sink, which starts at a random position and then moves throughout the simulation area according to the random waypoint mobility model [38]. The sensors deployed have four neighbors, except those at the borders. The different simulation parameters are those defined in Table 2, unless stated otherwise. The default inter-message period for MRLG RREQ packets was set to 1 second, while special announcements were generated once every 10 min.

We evaluate the performance when varying the number of sensors and the sink speed. The metrics used for the performance assessment are: the packet loss ratio, the latency and the routing load. To assess performance we compared the MRLG algorithm against a standard solution based on sink announcements, being CTP an example of such a protocol. With CTP, route discovery messages always propagate throughout the WSN to reach all nodes. The interval between consecutive route discovery messages was of 5 s since it offered the best trade-off between routing overhead and packet loss ratio when supporting sink mobility (for sink speeds up to 10 m/s).

### 8.1. Measuring the Performance When Varying the Number of Sources

In this first set of tests, we evaluated the behavior of the WSNs when increasing the number of traffic sources. Notice that, in a real world setting, the number of sources would represent sensor nodes reporting some critical condition to the sink. Thus, our purpose was to analyze the stability of the protocol, which should offer a nearly constant routing overhead despite the increase of source nodes.

Table 2 shows the most representative parameters used in the simulation experiments. The number of traffic sources varied from 1 to 40 nodes, while the total number of nodes was fixed at 200 nodes. The physical space where sensor nodes were deployed measured 140 × 140 m^2^, and the duration of each simulation was of 600 s.

**Figure 5.**
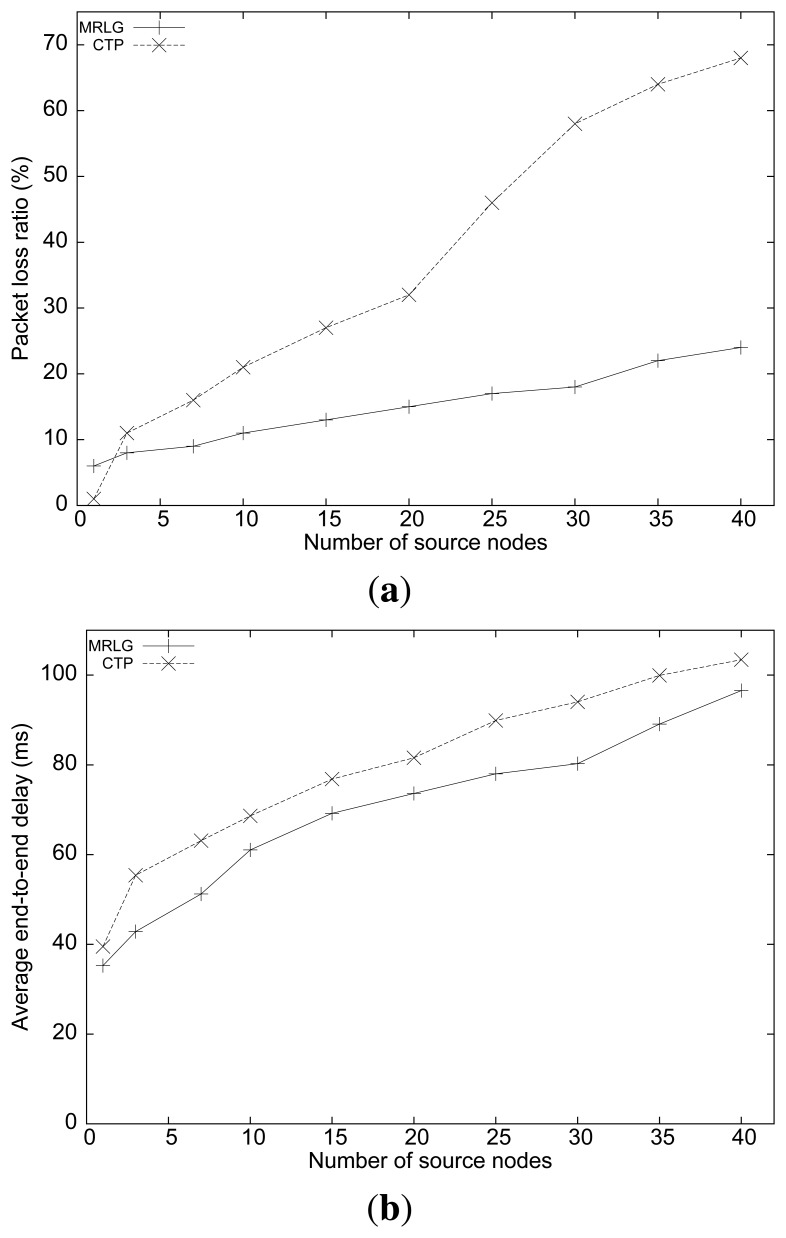
Varying the number of source nodes: (**a**) packet loss ratio, (**b**) end-to-end delay.

Figure 5(a) shows the packet loss ratio of the MRLG and the CTP algorithms, when varying the number of sources. We can observe that the loss ratio for the MRLG algorithm increased from 5% to 25% when the number of source nodes increased from 1 to 40, whereas for CTP the loss ratio increased dramatically (up to 70%) when the number of source nodes became 40. This difference is mostly due to contention with routing packets, as we show below.

Figure 5(b) shows the average end-to-end delay of both routing protocols. We can see that the average delay was lower for the MRLG protocol (approximately 10%). It can be observed that the differences in the performance of both algorithms in this experiment are quite noticeable, although not as significant as for the packet loss metric.

**Figure 6.**
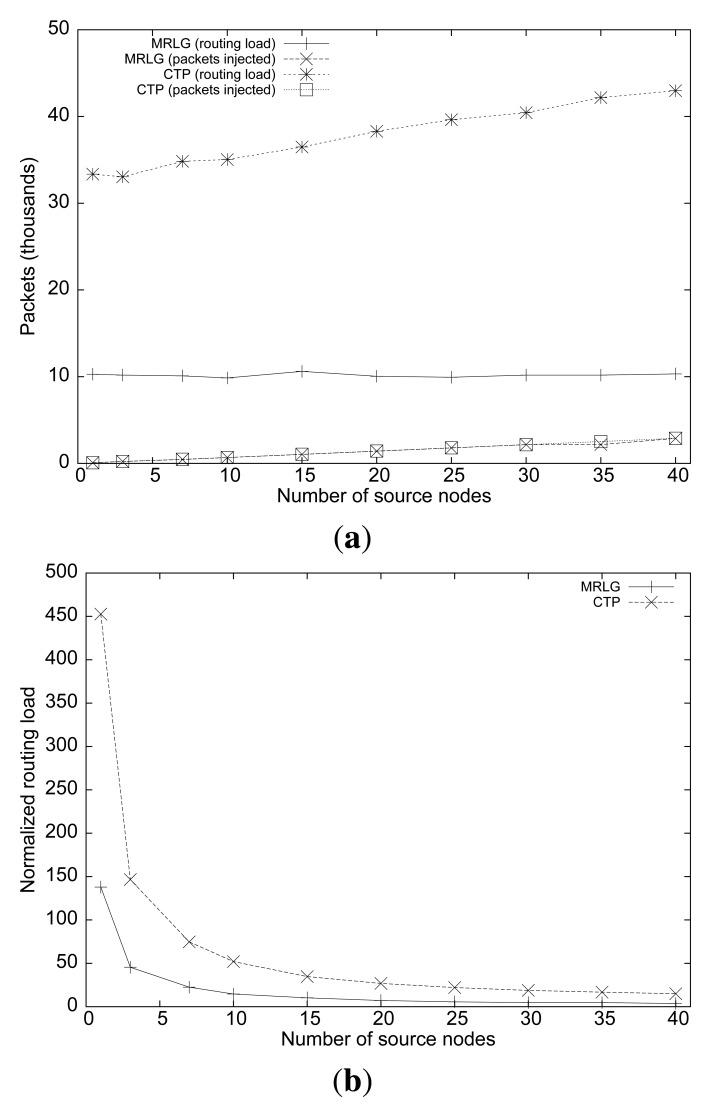
Varying the number of source nodes: (**a**) number of routing/injected packets, (**b**)normalized routing load.

Figure 6(a) shows the routing load performance of both algorithms. We can observe that, although the number of source nodes is increased, the routing load remained stable for the MRLG protocol (at approximately 10,000 packets), whereas for CTP the routing load increases slightly, being from three to four times larger than for MRLG. This is expected since both routing protocols create routes proactively, and independently of the actual number of sources present. Figure 6(b) shows the normalized routing load for both protocols, showing that most of the traffic overhead in the network is associated with the creation and maintenance of routes. Notice that sink mobility introduces significant topology updates, while nodes inject traffic at extremely low data rates.

Overall, the monitoring accuracy of a critical event (e.g., intruder tracking) depends both on packet loss ratio and delay for data messages. Thus, we can conclude that MRLG would allow a monitoring station to reconstruct the event pattern more accurately and in a more timely manner, thereby providing a better service.

### 8.2. Evaluating the Impact of Varying the Data Rate

In this section, we assess the performance differences between the CTP and the MRLG protocols when increasing the traffic injected in the WSN. Notice that larger data rates are associated with more frequent report messages generated by active sensors. This means that applications such as intruder tracking can obtain more precise location estimations due to more frequent updating. Thus, the purpose of this set of tests is to determine whether routing tasks are negatively affected by data traffic, and also to determine the threshold over which loss and delay become prohibitive for real-time tracking purposes.

Concerning the simulation parameters, we maintain the values defined in Table 2, except for the packet injection rate per-source, which we now vary from 0.05 to 1 pkt/s.

**Figure 7.**
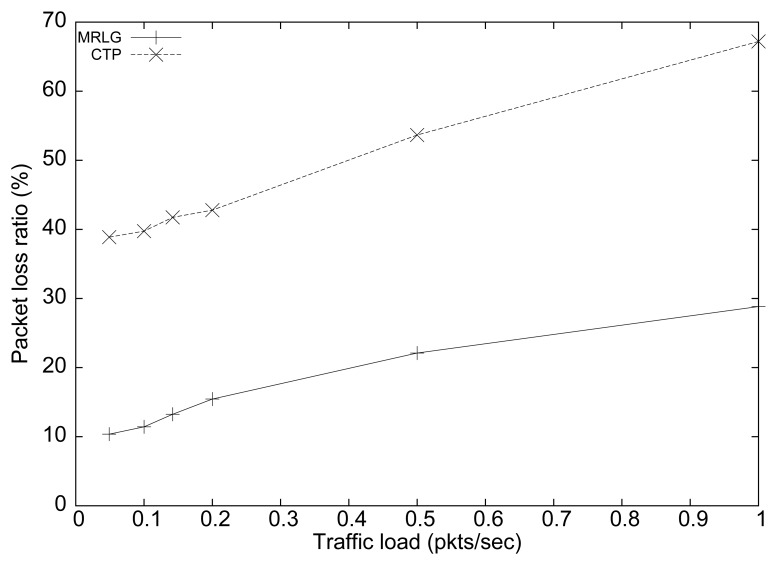
Packet loss ratio when varying load.

Figure 7 shows the performance of both protocols in terms of packet loss ratio. We can see that the percentage of loss for the CTP protocol was about 300% larger than for the MRLG protocol. As expected, both protocols showed an increasing trend for larger traffic loads, which was due to congestion. Again, the lower routing overhead introduced by MRLG leaves more room, in terms of bandwidth, for data packets, which explains the performance differences observed. Notice that the IEEE 802.15.4 technology supports only very low data rates (up to 250 kbit/s), meaning that bandwidth constraints impose very strong performance limitations.

**Figure 8.**
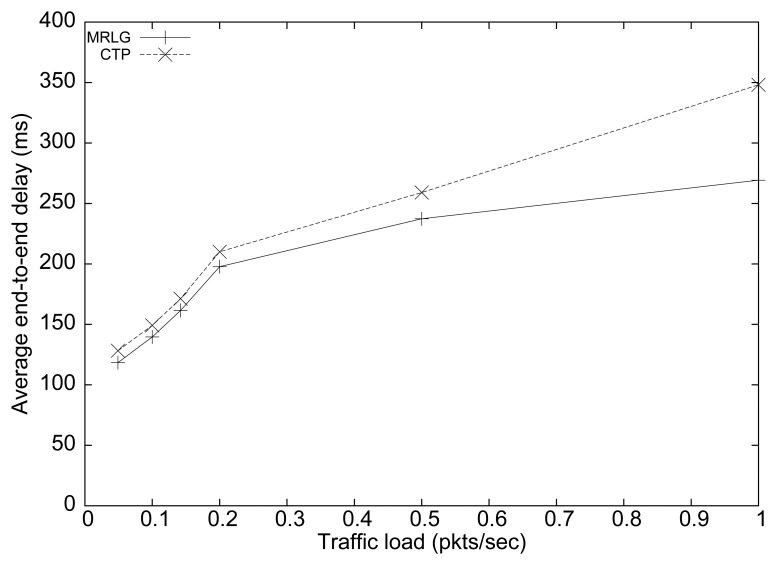
Average end-to-end delay when varying load.

Figure 8 shows the results obtained in terms of average end-to-end delay for both routing protocols. We can see that the delay differences were small for low load values. However, these differences increase for larger load values, reaching up to 80 ms for a traffic load of 1 packet per second (per source). In general, low delay values must be sought to favor a timely reaction of the sink to the reports received.

**Figure 9.**
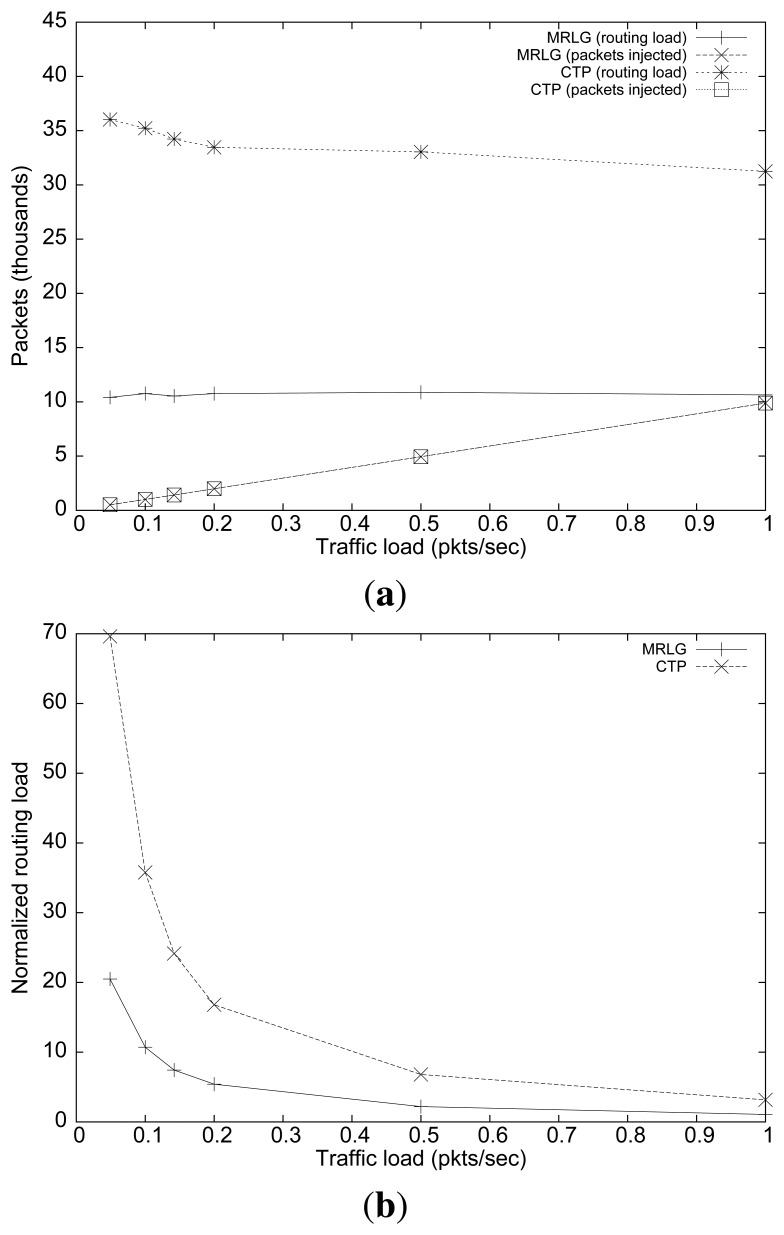
Varying the packet injection rate on a per-source basis: (**a**) number of routing/injected packets, (**b**) normalized routing load.

Figure 9(a) shows the number of routing and injected packets. In both cases, we find that the routing load remained mostly stable as the injected traffic increased, although CTP generated three times more packets than MRLG. This result manifests the effectiveness of the MRLG algorithm at minimizing the control overhead, while maintaining full effectiveness.

Figure 9(b) shows the normalized routing load. Again, we find substantial differences between both algorithms, especially for low traffic loads, reinforcing the aforementioned conclusions.

### 8.3. Assessing the Performance in Terms of Node Scalability

In the tests that follow, we vary the simulation area while maintaining the same number of source nodes, and the same number of packets injected per node. Our purpose is to evaluate the scalability of the routing algorithms when increasing the number of sensors in the WSN. In particular, we seek that the routing overhead increases as linearly as possible to avoid collapsing the WSN, while providing low route convergence times.

The number of nodes varies from 80 to 400, while the target area varies from 91 × 91 to 210 × 210 m^2^. Notice that we increase the area proportionally to the number of nodes to maintain the same sensor density.

**Figure 10.**
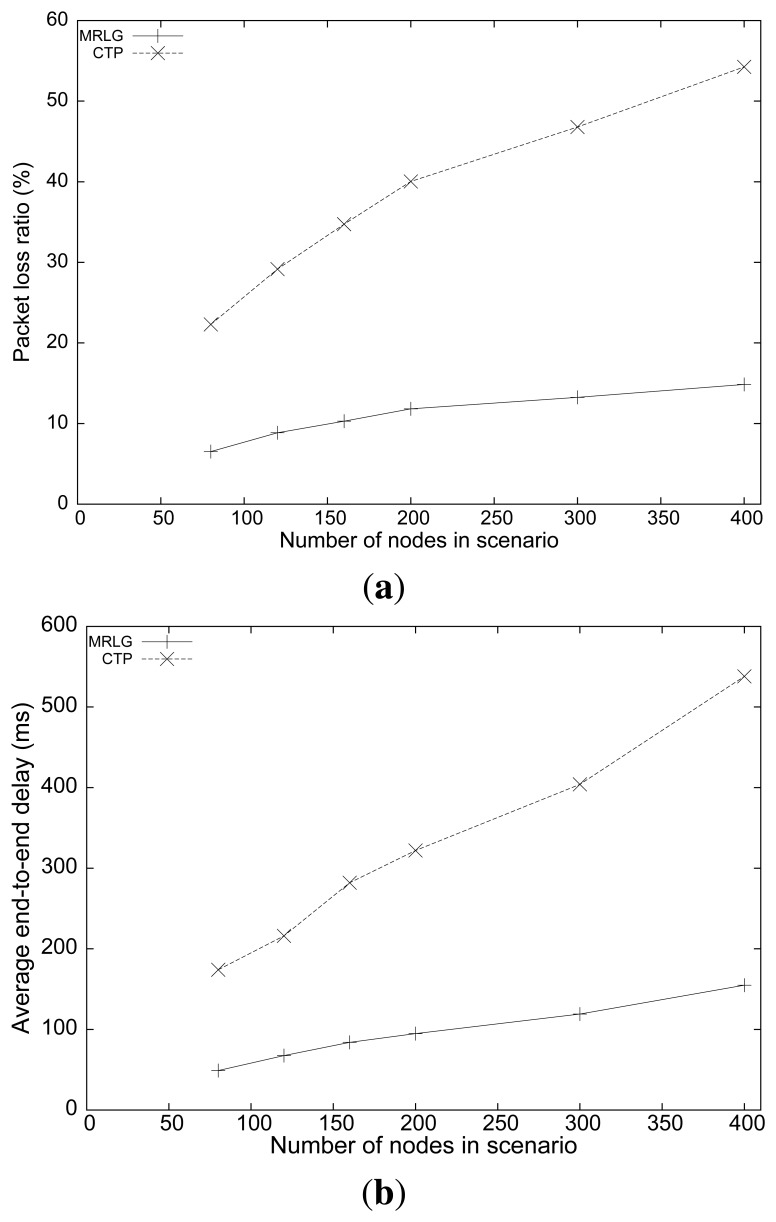
Varying the number of nodes per scenario: (**a**) packet loss ratio, (**b**) end-to-end delay.

Figure 10(a) shows the packet loss rate for the different scenarios analyzed. CTP showed a much larger loss rate than that of the MRLG protocol (between 3 and 4 times larger). In addition, we can observe that the MRLG protocol is more scalable, since the packet loss rate was larger for CTP than that for MRLG.

Figure 10(b) shows the average end-to-end delay. Notice that, while the delay for the MRLG protocol increased from 50 to 150 ms, in the case of CTP the delay values were larger, increasing from 170 to 530 ms. Such large delay values introduced by CTP clearly denote the problems experienced when supporting mobile sinks.

**Figure 11.**
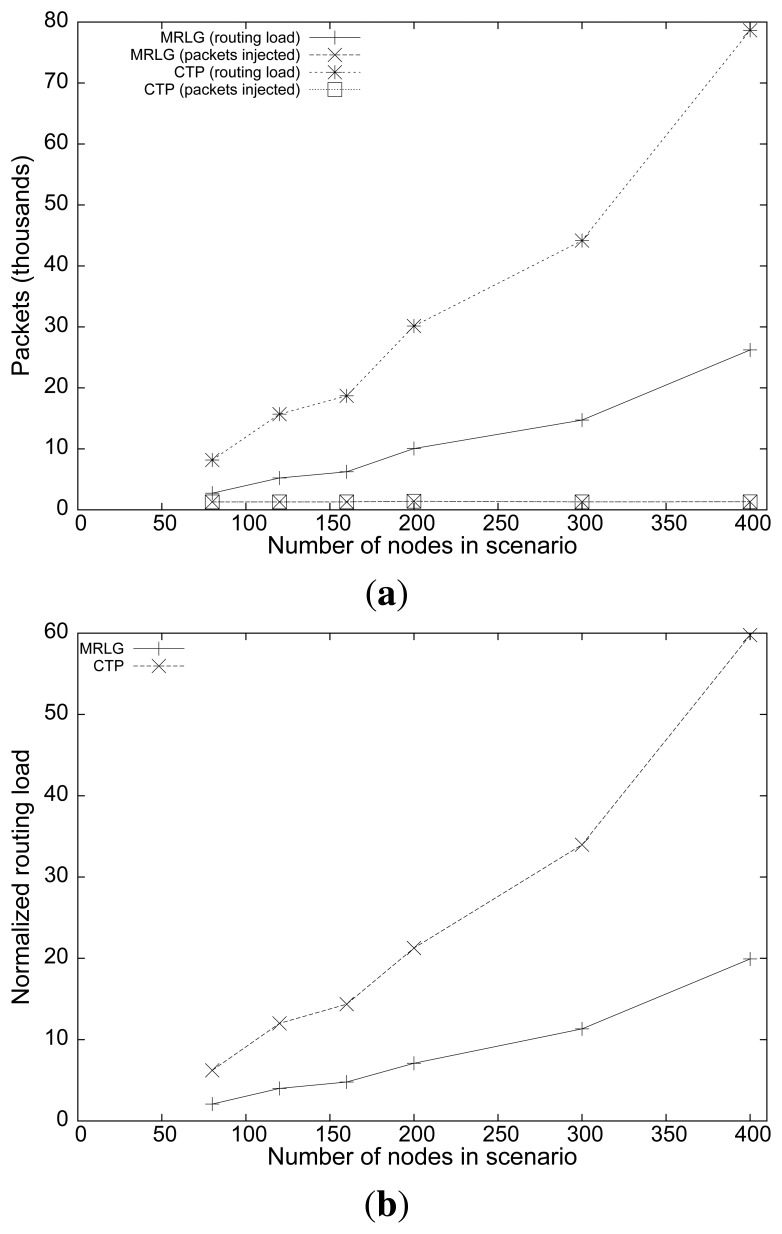
Varying the number of nodes per scenario: (**a**) number of routing/injected packets, (**b**) normalized routing load.

To gain further insight into this problem, Figure 11(a) shows the number of routing packets for both protocols, along with the number of injected packets. We observe that the routing load for MRLG (total number of control packets) increased linearly with the number of nodes in the scenario, as expected. However, CTP experienced a more than proportional increase, which became especially evident when the number of nodes was high. In fact, we found that the routing overhead results are intimately associated with the performance results of Figure 10, since the low channel capacity of IEEE 802.15.4 makes routing overhead a critical issue. This is evidenced by the normalized routing load results shown in Figure 11(b). Notice that most of the packets traveling along the network are routing related, meaning that minimizing the number of control packets has a clearly positive impact on both packet loss and delay. Once again, it is important to highlight that sink announcement based protocols, such as CTP, may suffer from scalability problems if no actions are taken to minimize the routing overhead introduced, like MRLG does.

### 8.4. Evaluating the Impact of Sink Speed

Finally, we present performance evaluation results for the MRLG protocol when varying the sink speed in the speed range of 1 to 10 m/s. The purpose of these experiments is to determine whether the routing protocols are able to offer low route convergence times. This requires maintaining delays and packet loss ratios at low values when speed increases; additionally, we aim at obtaining routing overhead values as low as possible.

**Figure 12.**
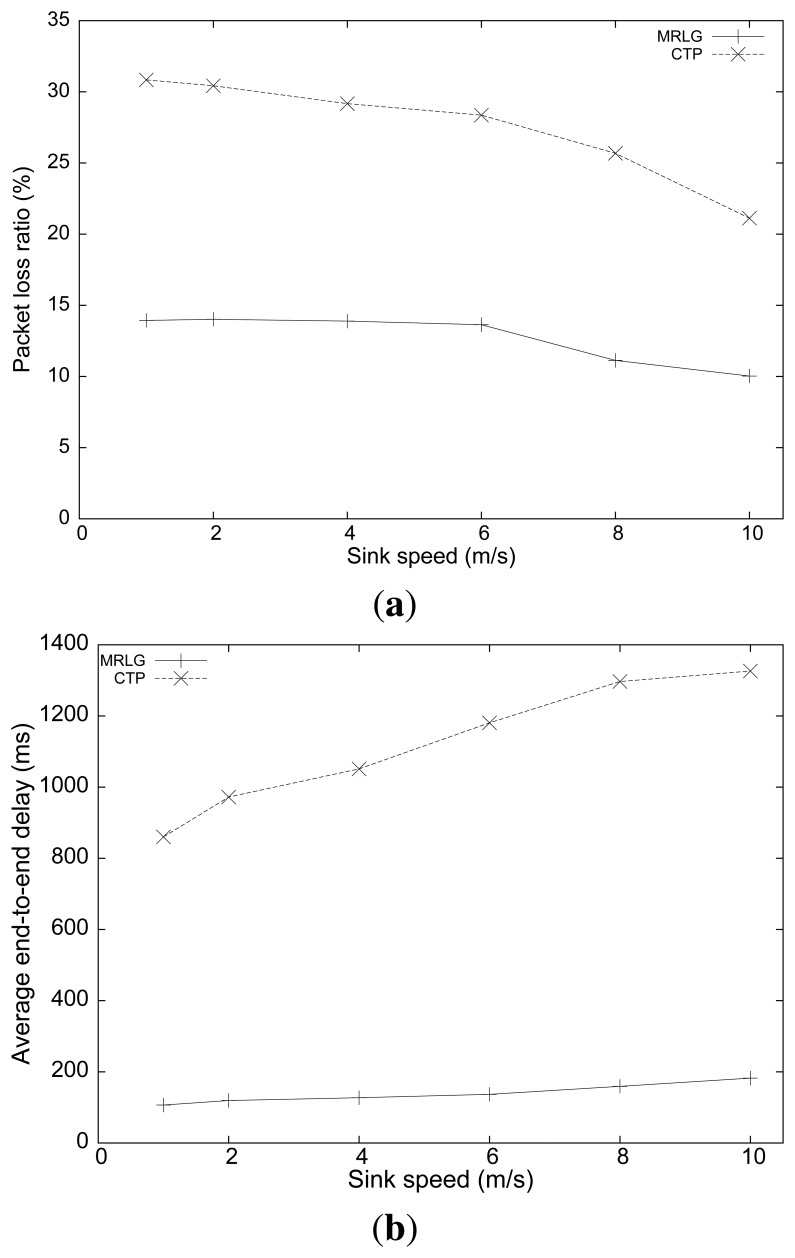
Varying the sink speed: (**a**) packet loss ratio, (**b**) end-to-end delay.

Figure 12(a) shows the packet loss rate obtained when we varied the sink speed. The loss rate for the MRLG protocol was quite smaller than that for the CTP protocol, showing a loss rate that was always maintained below 15%. It is also interesting to observe that the loss rate decreased when the sink speed increased, for both protocols. This behavior is due to the fact that the sink was more often near the center of the scenario, where the traffic requires less hops to reach the sink on average, thus experiencing less collision-related losses, which are more prone to occur for higher hop counts.

Figure 12(b) shows the results relative to end-to-end delay at different sink speeds. Now, we find that the average delay with MRLG was always maintained below 200 ms, whereas for CTP it ranged from 840 to 1350 ms. Such large delay values evidence that MRLG is able to perform adequately, and that high speeds do not represent any performance drawback (delay increases just slightly).

**Figure 13.**
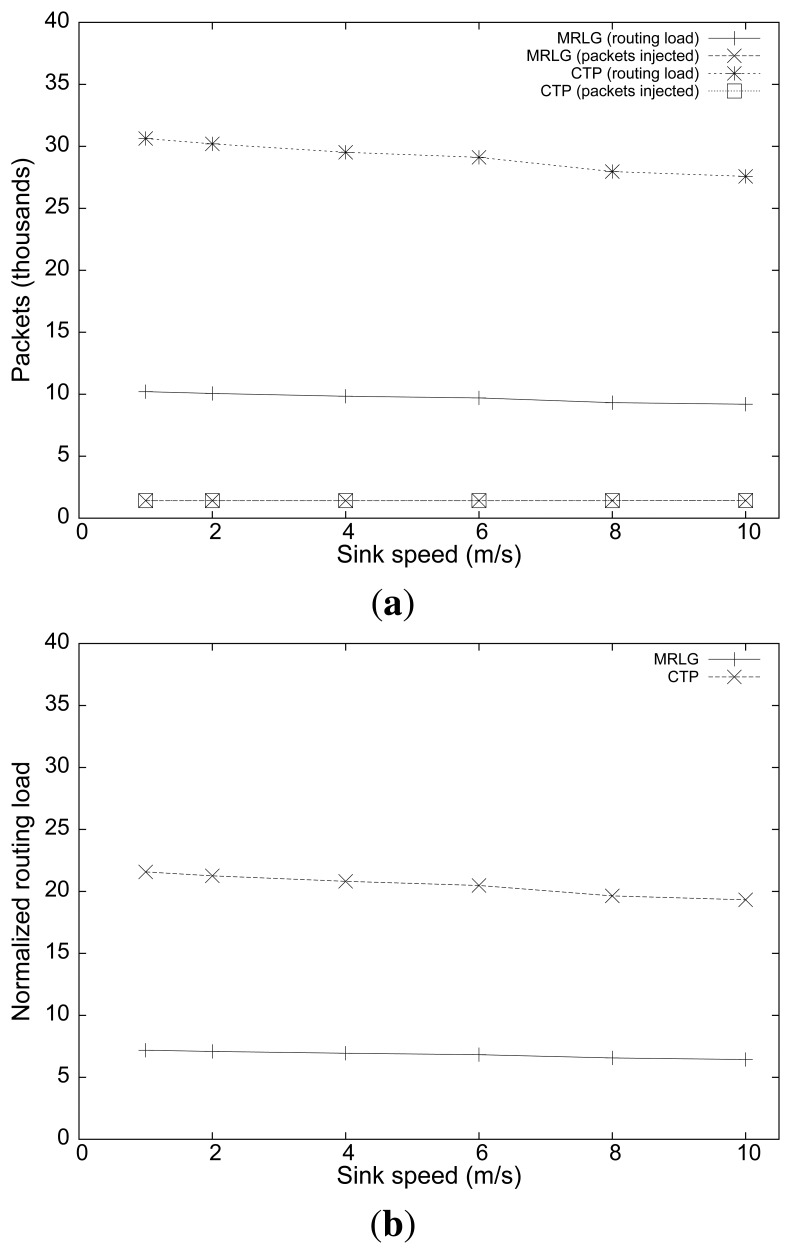
Varying the sink speed: (**a**) number of routing/injected packets, (**b**) normalized routing load.

Finally, Figure 13(a,b) shows the routing overhead and the normalized routing overhead. We now find that the routing overhead was maintained stable for both routing protocols, or was reduced slightly when the sink speed increased. These results evidence that, despite MRLG uses a much higher sink announcement (RREQ) rate to continuously detect local topology changes, it is able to achieve a better use of available resources, reducing the control overhead by up to 200%.

The results shown in this section allows us to conclude that, when combined with the IEEE 802.15.4 technology, MRLG is adequate for supporting applications requiring mobile sinks and low-delay data delivery. In fact, compared with a standard tree-based solution, the proposed MRLG protocol is able to drastically reduce routing overhead, which has a very positive impact in terms of packet losses and end-to-end delay.

## 9. Conclusions and Future Work

The design of a WSN is influenced by many factors including hardware constraints, transmission media, energy consumption, topology, scalability and fault tolerance. The significance of these factors increases in environments with several hundreds or thousands of sensor nodes. Additionally, the protocols and algorithms adopted must be efficient and scalable as well.

When targeting novel WSN applications, like real-time intruder tracking, information from different sources must be collected and processed as quickly as possible, to provide the sink with accurate information about the intruder location at all times. If, additionally, the sink needs to move along the WSN deployment scenario in an attempt to pursue and seize the intruder, the degree of complexity increases further, and the adopted routing protocol becomes critical.

In this paper, we introduced a comprehensive solution to achieve this goal when relying on cheap, binary detection sensors and the IEEE 802.15.4 technology. Our solution combines a fast intruder tracking algorithm with an efficient routing protocol (MRLG), to obtain the best performance. In particular, experimental results showed that, even at high mobility levels for both the intruder and the sink (fast running speeds), the proposed strategy allows the tracking error to be typically maintained below 10 m, even for highly irregular intruder mobility patterns. Additionally, we compare the proposed MRLG routing protocol against CTP, a known tree-based protocol, to further emphasize on the benefits of our proposal. Overall, we consider that the proposed solution is validated by the results obtained.

As future work, we plan to focus on mobile sink trajectory optimizations using more sophisticated scenarios that account for physical obstacles, combined with different sensor deployment strategies. We also plan to deploy and test the solution proposed in this paper in a real testbed, to further validate the high efficiency levels reported here.

## Figures and Tables

**Table 1.** Reference simulation parameters.

**Parameter**	**Default Value**
Number of Nodes	200
PHY/MAC	IEEE 802.15.4 / 2.4 GHz band
Traffic Type	CBR
Simulation Time	500 seconds
Simulation Area	200 × 100 m^2^
Sensor Topology	Grid
Routing Protocol	MRLG
Transmission Range	10 meters
Packet Size	50 bytes
Traffic Load	0.2 pkt/s
Intruder Speed	4 m/s
Sink Speed	4 m/s

**Table 2.** Default simulation parameters.

**Parameter**	**Default Value**
Number of nodes	200
PHY/MAC	IEEE 802.15.4 / 2.4 GHz band
Traffic Type	CBR
Simulation Time	600 seconds
Simulation Area	140 × 140 *m*^2^
Sensor Topology	Grid
Routing Protocol	MRLG/CTP
Transmission Range	10 meters
Packet Size	50 bytes
Number of Traffic sources	20
Traffic Load	0.2 pkt/s
Sink Speed	2 m/s
